# Supplementary material for: Impact of the COVID-19 pandemic on skin cancer diagnosis: A population-based study
Source: PLoS One. 2021 Mar 31;16(3):e0248492. doi: 10.1371/journal.pone.0248492 (PMC8011724; doi:10.1371/journal.pone.0248492)
Supplement: S1 Appendix — (DOCX) [file pone.0248492.s001.docx]

**S1 Appendix.**  Flow diagram for selection of study cohort of skin biopsy claims from January 7, 2019 to April 21, 2019

Skin biopsy claims in

January 7, 2019 to April 21, 2019

N=63,010 patients

Exclusive claims for identifying keratinocyte carcinoma and melanoma

N=61,684 patients

Adult residents in Ontario

N=59,807 patients

Residents with

eligible provincial coverage

N=57,744 patients

**Exclusion**

- History of anogenital cancer excision and diagnosis claims in a 5-year lookback period (1,326 patients)

**Exclusions**

- Missing age or sex (13 patients)
- ≤19 years of age (1,850 patients)
- Out-of-province residency (14 patients)

**Exclusion**

- Lapses in provincial medical coverage in a 5-year lookback and 1-year lookforward period (2,063 patients)
