# Supplementary material for: Impact of the COVID-19 pandemic on skin cancer diagnosis: A population-based study
Source: PLoS One. 2021 Mar 31;16(3):e0248492. doi: 10.1371/journal.pone.0248492 (PMC8011724; doi:10.1371/journal.pone.0248492)
Supplement: S2 Appendix — (DOCX) [file pone.0248492.s002.docx]

**S2 Appendix.** Patient characteristics for skin biopsy claims associated with a diagnosis of keratinocyte carcinoma for the first 15 weeks (starting on Monday) of 2019 and 2020.

| **Patient Characteristics** | **2019** | | | **2020** | | |
| --- | --- | --- | --- | --- | --- | --- |
|  | **Weeks 1-10 N=9,003** | **Weeks 11-15 N=4,731** | **Total N=13,734** | **Pre-COVID-19 Weeks 1-10 N=9,741** | **COVID-19 Weeks 11-15 N=1,035** | **Total N=10,776** |
| Age |  |  |  |  |  |  |
| Mean ± SD | 70.78 ± 13.38 | 71.37 ± 13.59 | 70.98 ± 13.45 | 71.23 ± 13.28 | 68.89 ± 13.01 | 71.01 ± 13.28 |
| Median (IQR) | 72 (62-81) | 73 (63-81) | 72 (62-81) | 72 (63-81) | 70 (61-78) | 72 (63-81) |
| Age (categorized) |  |  |  |  |  |  |
| 20-59 | 1,780 (19.77%) | 867 (18.33%) | 2,647 (19.27%) | 1,771 (18.18%) | 226 (21.84%) | 1,997 (18.53%) |
| 60-69 | 2,042 (22.68%) | 1,032 (21.81%) | 3,074 (22.38%) | 2,318 (23.80%) | 281 (27.15%) | 2,599 (24.12%) |
| 70-79 | 2,631 (29.22%) | 1,417 (29.95%) | 4,048 (29.47%) | 2,797 (28.71%) | 293 (28.31%) | 3,090 (28.67%) |
| 80+ | 2,550 (28.32%) | 1,415 (29.91%) | 3,965 (28.87%) | 2,855 (29.31%) | 235 (22.71%) | 3,090 (28.67%) |
| Sex |  |  |  |  |  |  |
| Female | 4,054 (45.03%) | 2,141 (45.25%) | 6,195 (45.11%) | 4,434 (45.52%) | 408 (39.42%) | 4,842 (44.93%) |
| Male | 4,949 (54.97%) | 2,590 (54.75%) | 7,539 (54.89%) | 5,307 (54.48%) | 627 (60.58%) | 5,934 (55.07%) |
| Income quintiles^1^ |  |  |  |  |  |  |
| 1 | 1,295 (14.38%) | 694 (14.67%) | 1,989 (14.48%) | 1,322 (13.57%) | 123 (11.88%) | 1,445 (13.41%) |
| 2 | 1,702 (18.90%) | 826 (17.46%) | 2,528 (18.41%) | 1,765 (18.12%) | 170 (16.43%) | 1,935 (17.96%) |
| 3 | 1,780 (19.77%) | 934 (19.74%) | 2,714 (19.76%) | 1,896 (19.46%) | 209 (20.19%) | 2,105 (19.53%) |
| 4 | 1,814 (20.15%) | 971 (20.52%) | 2,785 (20.28%) | 2,063 (21.18%) | 228 (22.03%) | 2,291 (21.26%) |
| 5 | 2,397 (26.62%) | 1,302 (27.52%) | 3,699 (26.93%) | 2,679 (27.50%) | 304 (29.37%) | 2,983 (27.68%) |
| Rurality Index for Ontario^1^ |  |  |  |  |  |  |
| Urban (0-9) | 5,784 (64.25%) | 3,068 (64.85%) | 8,852 (64.45%) | 6,359 (65.28%) | 640 (61.84%) | 6,999 (64.95%) |
| Suburban (10-39) | 2,294 (25.48%) | 1,235 (26.10%) | 3,529 (25.70%) | 2,554 (26.22%) | 278 (26.86%) | 2,832 (26.28%) |
| Rural (40+) | 882 (9.80%) | 404 (8.54%) | 1,286 (9.36%) | 782 (8.03%) | 107 (10.34%) | 889 (8.25%) |
| Place of residence (LHIN) |  |  |  |  |  |  |
| 01 | 531 (5.90%) | 280 (5.92%) | 811 (5.91%) | 611 (6.27%) | 73 (7.05%) | 684 (6.35%) |
| 02 | 1,108 (12.31%) | 512 (10.82%) | 1,620 (11.80%) | 1,144 (11.74%) | 76 (7.34%) | 1,220 (11.32%) |
| 03 | 641 (7.12%) | 335 (7.08%) | 976 (7.11%) | 705 (7.24%) | 71 (6.86%) | 776 (7.20%) |
| 04 | 1,302 (14.46%) | 695 (14.69%) | 1,997 (14.54%) | 1,234 (12.67%) | 140 (13.53%) | 1,374 (12.75%) |
| 05 | 232 (2.58%) | 142 (3.00%) | 374 (2.72%) | 281 (2.88%) | 19 (1.84%) | 300 (2.78%) |
| 06 | 611 (6.79%) | 341 (7.21%) | 952 (6.93%) | 745 (7.65%) | 91 (8.79%) | 836 (7.76%) |
| 07 | 724 (8.04%) | 395 (8.35%) | 1,119 (8.15%) | 778 (7.99%) | 70 (6.76%) | 848 (7.87%) |
| 08 | 714 (7.93%) | 407 (8.60%) | 1,121 (8.16%) | 877 (9.00%) | 71 (6.86%) | 948 (8.80%) |
| 09 | 889 (9.87%) | 482 (10.19%) | 1,371 (9.98%) | 978 (10.04%) | 90 (8.70%) | 1,068 (9.91%) |
| 10 | 509 (5.65%) | 237 (5.01%) | 746 (5.43%) | 542 (5.56%) | 78 (7.54%) | 620 (5.75%) |
| 11 | 942 (10.46%) | 530 (11.20%) | 1,472 (10.72%) | 1,035 (10.63%) | 114 (11.01%) | 1,149 (10.66%) |
| 12 | 320 (3.55%) | 155 (3.28%) | 475 (3.46%) | 331 (3.40%) | 50 (4.83%) | 381 (3.54%) |
| 13 | 371 (4.12%) | 171 (3.61%) | 542 (3.95%) | 366 (3.76%) | 76 (7.34%) | 442 (4.10%) |
| 14 | 109 (1.21%) | 49 (1.04%) | 158 (1.15%) | 114 (1.17%) | 16 (1.55%) | 130 (1.21%) |
| Elixhauser comorbidity index^2^ |  |  |  |  |  |  |
| 0 | 6,487 (72.05%) | 3,395 (71.76%) | 9,882 (71.95%) | 7,061 (72.49%) | 784 (75.75%) | 7,845 (72.80%) |
| 1-2 | 1,765 (19.60%) | 919 (19.43%) | 2,684 (19.54%) | 1,823 (18.71%) | 166 (16.04%) | 1,989 (18.46%) |
| 3+ | 751 (8.34%) | 417 (8.81%) | 1,168 (8.50%) | 857 (8.80%) | 85 (8.21%) | 942 (8.74%) |
| Physician specialty billing biopsy claims |  |  |  |  |  |  |
| Dermatology | 5,271 (58.55%) | 2,879 (60.85%) | 8,150 (59.34%) | 5,818 (59.73%) | 546 (52.75%) | 6,364 (59.06%) |
| GP/FP | 1,361 (15.12%) | 717 (15.16%) | 2,078 (15.13%) | 1,614 (16.57%) | 164 (15.85%) | 1,778 (16.50%) |
| General surgery | 373 (4.14%) | 189 (3.99%) | 562 (4.09%) | 324 (3.33%) | 48 (4.64%) | 372 (3.45%) |
| Plastic surgery | 1,306 (14.51%) | 648 (13.70%) | 1,954 (14.23%) | 1,292 (13.26%) | 193 (18.65%) | 1,485 (13.78%) |
| Otolaryngology | 602 (6.69%) | 243 (5.14%) | 845 (6.15%) | 584 (6.00%) | 73 (7.05%) | 657 (6.10%) |
| Other | 90 (1.00%) | 55 (1.16%) | 145 (1.06%) | 109 (1.12%) | 11 (1.06%) | 120 (1.11%) |

**Abbreviations:**

SD: standard deviation, IQR: interquartile range, LHIN: Local Health Integration Network, GP/FP: general practitioner/family practitioner.

**Notes:**

1. Column percentages may not sum to 100% due to missing data.

2. Diagnostic codes for cancer metastasis or solid tumor without metastasis were excluded from the comorbidity score.
