# Supplementary material for: Impact of the COVID-19 pandemic on skin cancer diagnosis: A population-based study
Source: PLoS One. 2021 Mar 31;16(3):e0248492. doi: 10.1371/journal.pone.0248492 (PMC8011724; doi:10.1371/journal.pone.0248492)
Supplement: S3 Appendix — (DOCX) [file pone.0248492.s003.docx]

**S3 Appendix.** Relative risks for comparing skin biopsy claims associated with a diagnosis of keratinocyte carcinoma between COVID-19 (Weeks 11-15) and pre-COVID-19 (Weeks 1-10) periods for the first 15 weeks (starting on Monday) of 2020.

| **Patient Characteristics** | **Unadjusted Analysis** | | **Adjusted Analysis^1^** | |
| --- | --- | --- | --- | --- |
|  | **RR (95% CI)** | **P-Value** | **RR (95% CI)** | **P-Value** |
| Age (categorized) |  |  |  |  |
| 20-59 | Reference |  | Reference |  |
| 60-69 | 0.95 (0.81-1.13) | 0.576 | 0.93 (0.79-1.10) | 0.420 |
| 70-79 | 0.84 (0.71-0.99) | 0.037 | 0.83 (0.70-0.99) | 0.033 |
| 80+ | 0.68 (0.57-0.81) | <0.001 | 0.69 (0.57-0.83) | <0.001 |
| Sex |  |  |  |  |
| Female | Reference |  | Reference |  |
| Male | 1.25 (1.11-1.41) | <0.001 | 1.25 (1.11-1.41) | <0.001 |
| Income quintiles |  |  |  |  |
| 1 | Reference |  | Reference |  |
| 2 | 1.04 (0.83-1.30) | 0.718 | 1.06 (0.85-1.32) | 0.616 |
| 3 | 1.16 (0.93-1.43) | 0.186 | 1.15 (0.93-1.42) | 0.199 |
| 4 | 1.17 (0.95-1.45) | 0.142 | 1.17 (0.95-1.44) | 0.149 |
| 5 | 1.21 (0.99-1.47) | 0.068 | 1.22 (1.00-1.50) | 0.053 |
| Rurality Index for Ontario |  |  |  |  |
| Urban (0-9) | 0.76 (0.63-0.92) | 0.005 | 0.85 (0.68-1.07) | 0.158 |
| Suburban (10-39) | 0.82 (0.66-1.01) | 0.057 | 0.82 (0.65-1.04) | 0.103 |
| Rural (40+) | Reference |  | Reference |  |
| Place of residence (LHIN) |  |  |  |  |
| 01 | 0.85 (0.63-1.14) | 0.278 | 0.82 (0.61-1.10) | 0.191 |
| 02 | 0.49 (0.36-0.66) | <0.001 | 0.49 (0.36-0.67) | <0.001 |
| 03 | 0.73 (0.54-0.98) | 0.039 | 0.74 (0.54-1.01) | 0.060 |
| 04 | 0.80 (0.62-1.04) | 0.097 | 0.86 (0.65-1.12) | 0.261 |
| 05 | 0.50 (0.31-0.81) | 0.005 | 0.50 (0.30-0.82) | 0.006 |
| 06 | 0.86 (0.65-1.15) | 0.307 | 0.94 (0.69-1.28) | 0.684 |
| 07 | 0.66 (0.48-0.89) | 0.007 | 0.73 (0.52-1.01) | 0.057 |
| 08 | 0.59 (0.44-0.81) | <0.001 | 0.66 (0.48-0.91) | 0.010 |
| 09 | 0.67 (0.50-0.89) | 0.006 | 0.66 (0.50-0.89) | 0.006 |
| 10 | Reference |  | Reference |  |
| 11 | 0.79 (0.60-1.03) | 0.086 | 0.82 (0.62-1.09) | 0.177 |
| 12 | 1.05 (0.75-1.46) | 0.793 | 1.00 (0.71-1.42) | 0.991 |
| 13 | 1.30 (0.96-1.75) | 0.090 | 1.24 (0.91-1.69) | 0.173 |
| 14 | 0.99 (0.59-1.66) | 0.971 | 0.82 (0.49-1.40) | 0.471 |
| Elixhauser comorbidity index^2^ |  |  |  |  |
| 0 | Reference |  | Reference |  |
| 1-2 | 0.84 (0.71-0.99) | 0.033 | 0.87 (0.74-1.03) | 0.112 |
| 3+ | 0.90 (0.73-1.12) | 0.340 | 0.98 (0.78-1.22) | 0.827 |
| Physician specialty billing biopsy claims |  |  |  |  |
| Dermatology | 0.94 (0.53-1.65) | 0.818 | 0.99 (0.55-1.76) | 0.963 |
| GP/FP | 0.99 (0.55-1.77) | 0.973 | 1.07 (0.60-1.93) | 0.818 |
| General surgery | 1.40 (0.75-2.61) | 0.289 | 1.42 (0.76-2.65) | 0.275 |
| Plastic surgery | 1.42 (0.79-2.53) | 0.238 | 1.49 (0.83-2.67) | 0.184 |
| Otolaryngology | 1.20 (0.65-2.19) | 0.560 | 1.26 (0.69-2.32) | 0.451 |
| Other | Reference |  | Reference |  |

**Abbreviations:**

RR: relative risk, CI: confidence interval, LHIN: Local Health Integration Network, GP/FP: general practitioner/family practitioner.

**Notes:**

1. The full adjusted model contains all variables in the unadjusted analyses.

2. Diagnostic codes for cancer metastasis or solid tumor without metastasis were excluded from the comorbidity score.
