# Supplementary material for: Impact of the COVID-19 pandemic on skin cancer diagnosis: A population-based study
Source: PLoS One. 2021 Mar 31;16(3):e0248492. doi: 10.1371/journal.pone.0248492 (PMC8011724; doi:10.1371/journal.pone.0248492)
Supplement: S4 Appendix — (DOCX) [file pone.0248492.s004.docx]

**S4 Appendix.** Patient characteristics for skin biopsy claims associated with a diagnosis of melanoma for the first 15 weeks (starting on Monday) of 2019 and 2020.

| **Patient Characteristics** | **2019** | | | **2020** | | |
| --- | --- | --- | --- | --- | --- | --- |
|  | **Weeks 1-10 N=587** | **Weeks 11-15 N=323** | **Total N=910** | **Pre-COVID-19 Weeks 1-10 N=640** | **COVID-19 Weeks 11-15 N=96** | **Total**  **N=736** |
| Age |  |  |  |  |  |  |
| Mean ± SD | 64.94 ± 15.20 | 64.54 ± 15.91 | 64.80 ± 15.45 | 65.75 ± 15.42 | 65.44 ± 15.12 | 65.71 ± 15.37 |
| Median (IQR) | 67 (56-76) | 67 (55-77) | 67 (56-76) | 69 (57-77) | 69 (59-76) | 69 (57-77) |
| Age (categorized) |  |  |  |  |  |  |
| 20-59 | 177 (30.15%) | 107 (33.13%) | 284 (31.21%) | 183 (28.59%) | 25 (26.04%) | 208 (28.26%) |
| 60-69 | 158 (26.92%) | 75 (23.22%) | 233 (25.60%) | 152 (23.75%) | 27 (28.13%) | 179 (24.32%) |
| 70-79 | 161 (27.43%) | 81 (25.08%) | 242 (26.59%) | 188 (29.38%) | 31 (32.29%) | 219 (29.76%) |
| 80+ | 91 (15.50%) | 60 (18.58%) | 151 (16.59%) | 117 (18.28%) | 13 (13.54%) | 130 (17.66%) |
| Sex |  |  |  |  |  |  |
| Female | 291 (49.57%) | 152 (47.06%) | 443 (48.68%) | 315 (49.22%) | 39 (40.63%) | 354 (48.10%) |
| Male | 296 (50.43%) | 171 (52.94%) | 467 (51.32%) | 325 (50.78%) | 57 (59.38%) | 382 (51.90%) |
| Income quintiles^1^ |  |  |  |  |  |  |
| 1 | 76 (12.95%) | 55 (17.03%) | 131 (14.40%) | 98 (15.31%) | 9 (9.38%) | 107 (14.54%) |
| 2 | 118 (20.10%) | 57 (17.65%) | 175 (19.23%) | 106 (16.56%) | 13 (13.54%) | 119 (16.17%) |
| 3 | 102 (17.38%) | 70 (21.67%) | 172 (18.90%) | 136 (21.25%) | 22 (22.92%) | 158 (21.47%) |
| 4 | 139 (23.68%) | 62 (19.20%) | 201 (22.09%) | 139 (21.72%) | 17 (17.71%) | 156 (21.20%) |
| 5 | 152 (25.89%) | 79 (24.46%) | 231 (25.38%) | 159 (24.84%) | 35 (36.46%) | 194 (26.36%) |
| Rurality Index for Ontario^1^ |  |  |  |  |  |  |
| Urban (0-9) | 281 (47.87%) | 145 (44.89%) | 426 (46.81%) | 291 (45.47%) | 42 (43.75%) | 333 (45.24%) |
| Suburban (10-39) | 238 (40.55%) | 143 (44.27%) | 381 (41.87%) | 288 (45.00%) | 34 (35.42%) | 322 (43.75%) |
| Rural (40+) | 63 (10.73%) | 34 (10.53%) | 97 (10.66%) | 58 (9.06%) | 17 (17.71%) | 75 (10.19%) |
| Place of residence (LHIN) |  |  |  |  |  |  |
| West (01-04) | 263 (44.80%) | 141 (43.65%) | 404 (44.40%) | 271 (42.34%) | 31 (32.29%) | 302 (41.03%) |
| Central (05, 06, 08, 12), or Toronto (07) | 108 (18.40%) | 63 (19.50%) | 171 (18.79%) | 129 (20.16%) | 23 (23.96%) | 152 (20.65%) |
| East (09-11) | 163 (27.77%) | 97 (30.03%) | 260 (28.57%) | 184 (28.75%) | 26 (27.08%) | 210 (28.53%) |
| North (13-14) | 53 (9.03%) | 22 (6.81%) | 75 (8.24%) | 56 (8.75%) | 16 (16.67%) | 72 (9.78%) |
| Elixhauser comorbidity index^2^ |  |  |  |  |  |  |
| 0 | 439 (74.79%) | 243 (75.23%) | 682 (74.95%) | 490 (76.56%) | 64 (66.67%) | 554 (75.27%) |
| 1-2 | 104 (17.72%) | 58 (17.96%) | 162 (17.80%) | 107 (16.72%) | 21 (21.88%) | 128 (17.39%) |
| 3+ | 44 (7.50%) | 22 (6.81%) | 66 (7.25%) | 43 (6.72%) | 11 (11.46%) | 54 (7.34%) |
| Physician specialty billing biopsy claims |  |  |  |  |  |  |
| Dermatology | 208 (35.43%) | 105 (32.51%) | 313 (34.40%) | 232 (36.25%) | 33 (34.38%) | 265 (36.01%) |
| GP/FP | 116 (19.76%) | 75 (23.22%) | 191 (20.99%) | 145 (22.66%) | 16 (16.67%) | 161 (21.88%) |
| General surgery | 144 (24.53%) | 78 (24.15%) | 222 (24.40%) | 150 (23.44%) | 18 (18.75%) | 168 (22.83%) |
| Plastic surgery | 79 (13.46%) | 42 (13.00%) | 121 (13.30%) | 74 (11.56%) | 15 (15.63%) | 89 (12.09%) |
| Otolaryngology, or other | 40 (6.81%) | 23 (7.12%) | 63 (6.92%) | 39 (6.09%) | 14 (14.58%) | 53 (7.20%) |

**Abbreviations:**

SD: standard deviation, IQR: interquartile range, LHIN: Local Health Integration Network, GP/FP: general practitioner/family practitioner.

**Notes:**

1. Column percentages may not sum to 100% due to missing data.

2. Diagnostic codes for cancer metastasis or solid tumor without metastasis were excluded from the comorbidity score.
