# Supplementary material for: Impact of the COVID-19 pandemic on skin cancer diagnosis: A population-based study
Source: PLoS One. 2021 Mar 31;16(3):e0248492. doi: 10.1371/journal.pone.0248492 (PMC8011724; doi:10.1371/journal.pone.0248492)
Supplement: S5 Appendix — (DOCX) [file pone.0248492.s005.docx]

**S5 Appendix.** Relative risks for comparing skin biopsy claims associated with a diagnosis of melanoma between COVID-19 (Weeks 11-15) and pre-COVID-19 (Weeks 1-10) periods for the first 15 weeks (starting on Monday) of 2020.

| **Patient Characteristics** | **Unadjusted Analysis** | | **Adjusted Analysis^1^** | |
| --- | --- | --- | --- | --- |
|  | **RR (95% CI)** | **P-Value** | **RR (95% CI)** | **P-Value** |
| Age (categorized) |  |  |  |  |
| 20-59 | Reference |  | Reference |  |
| 60-69 | 1.26 (0.75-2.11) | 0.380 | 1.05 (0.62-1.77) | 0.855 |
| 70-79 | 1.19 (0.72-1.96) | 0.504 | 0.94 (0.55-1.59) | 0.807 |
| 80+ | 0.86 (0.45-1.63) | 0.639 | 0.70 (0.36-1.36) | 0.291 |
| Sex |  |  |  |  |
| Female | Reference |  | Reference |  |
| Male | 1.30 (0.89-1.92) | 0.178 | 1.15 (0.77-1.72) | 0.486 |
| Income quintiles |  |  |  |  |
| 1 | Reference |  | Reference |  |
| 2 | 1.62 (0.67-3.91) | 0.281 | 1.63 (0.68-3.90) | 0.275 |
| 3 | 2.07 (0.92-4.67) | 0.080 | 1.98 (0.88-4.44) | 0.097 |
| 4 | 1.62 (0.70-3.77) | 0.264 | 1.70 (0.73-3.94) | 0.216 |
| 5 | 2.62 (1.20-5.70) | 0.015 | 2.59 (1.20-5.60) | 0.016 |
| Rurality Index for Ontario |  |  |  |  |
| Urban (0-9) | 0.56 (0.34-0.92) | 0.023 | 0.56 (0.30-1.04) | 0.065 |
| Suburban (10-39) | 0.47 (0.28-0.79) | 0.004 | 0.54 (0.30-0.98) | 0.042 |
| Rural (40+) | Reference |  | Reference |  |
| Place of residence (LHIN) |  |  |  |  |
| West (01-04) | 0.83 (0.51-1.36) | 0.462 | 0.67 (0.38-1.16) | 0.153 |
| Central (05, 06, 08, 12), or Toronto (07) | 1.23 (0.73-2.07) | 0.435 | 0.84 (0.45-1.56) | 0.579 |
| East (09-11) | Reference |  | Reference |  |
| North (13-14) | 1.54 (0.84-2.83) | 0.161 | 1.13 (0.57-2.24) | 0.735 |
| Elixhauser comorbidity index^2^ |  |  |  |  |
| 0 | Reference |  | Reference |  |
| 1-2 | 1.47 (0.93-2.33) | 0.096 | 1.38 (0.88-2.17) | 0.163 |
| 3+ | 1.83 (1.03-3.26) | 0.040 | 2.01 (1.09-3.71) | 0.025 |
| Physician specialty billing biopsy claims |  |  |  |  |
| Dermatology | 0.45 (0.26-0.79) | 0.005 | 0.52 (0.30-0.92) | 0.024 |
| GP/FP | 0.35 (0.18-0.67) | 0.002 | 0.36 (0.17-0.73) | 0.005 |
| General surgery | 0.38 (0.20-0.71) | 0.003 | 0.52 (0.26-1.03) | 0.062 |
| Plastic surgery | 0.63 (0.33-1.19) | 0.153 | 0.75 (0.40-1.43) | 0.382 |
| Otolaryngology, or other | Reference |  | Reference |  |

**Abbreviations:**

RR: relative risk, CI: confidence interval, LHIN: Local Health Integration Network, GP/FP: general practitioner/family practitioner.

**Notes:**

1. The full adjusted model contains all variables in the unadjusted analyses.

2. Diagnostic codes for cancer metastasis or solid tumor without metastasis were excluded from the comorbidity score.
