# Supplementary material for: Impact of the COVID-19 pandemic on skin cancer diagnosis: A population-based study
Source: PLoS One. 2021 Mar 31;16(3):e0248492. doi: 10.1371/journal.pone.0248492 (PMC8011724; doi:10.1371/journal.pone.0248492)
Supplement: Attachment — Submitted filename: Response to Reviewers FINAL.docx [file pone.0248492.s006.docx]

PONE-D-20-37376

Impact of the COVID-19 pandemic on skin cancer diagnosis: a population-based study

PLOS ONE

**General comments**

Thank-you to the reviewers and editorial team for reviewing our manuscript. We have revised our manuscript to follow formatting and reporting criteria for PLOS ONE. Below are our specific comments to the Academic Editor and Reviewers.

**Academic Editor Comment**

**Even if both reviewers found the manuscript interesting, some additional evaluation and comments on the obtained data would be useful to improve the manuscript's relevance.**

Thank-you. We have revised our manuscript to provide more up to date data on skin biopsies. We note that at the time of submission of our manuscript, our report included the most up-to-date data available at the time. More data has become available during the time taken to process our article and review it, and we are pleased to be able to provide that as part of our manuscript.

We provide further details on the new content in response to Reviewer #2. We have incorporated content on the new data into our results, figures and discussion. Unfortunately, stage and incidence data was not available to us, and we emphasize this limitation in our discussion.

**Reviewer #1: Very well written article. It is highlighting a major health concern we have encountered during the current pandemic. Hence I think the topic is very relevant. I think similar studies if undertaken in other countries would show the same decline during the cOVID-19 pandemic. Hence I think its results can be generalized to other countries and populations. Authors have used technically sound data analysis and interpretation.**

Thank-you Reviewer #1. We appreciate the time you have taken to review our manuscript and your constructive comments.

**Reviewer #2: This study presents an interesting observation concerning a drop in skin biopsy for cancer during the COVID pandemic. These results are largely expected based on restrictions on access to heath care during the acute phase of the pandemic. It is not surprising that the drop affected older people more than younger ones, but it is less clearly interpretable why it affected women more than men. How the delay translated into more advanced cancer stage, deaths or complications due to cancer was not explored. This is a major limitation of the study. In addition, no information was provided about what happened in the period of time subsequent to the pandemic more acute phase (namely, after May 2020): was an increase in the number of skin biopsies observed compared to the expected numbers to compensate for the reduction of interventions during the acute phase? what was the profile of patients treated thereafter?
In my opinion, in a highly dynamic situation such as the pandemic, a more comprehensive view on trends and attitudes should be obtained to better understand how healthcare changes affect outcomes**

Thank-you Reviewer #2. We appreciate the time you have taken to review our manuscript and your constructive comments.

For our submission, we focused on the early lockdown from the first wave of COVID-19 in Ontario (2020, weeks 11-15) for two specific reasons. First, we wanted to study a discrete time period, in order to rigorously document the impact of COVID-19 on skin biopsies at the height of lockdown measures. Secondly, as we wished our study to be both rigorous and timely, we limited our study period to that for which we had sufficiently complete data. We note that at the time of submission of our manuscript, and even when we obtained the data for this revision, there was no provincial cancer registry data available for the year 2020, due largely to the impact of COVID-19 an all aspects of the health care system and research services in Ontario.

Though we are restricted in our ability to utilize cancer registry data on cancer incidence, stage or survival for this project, we were able to obtain sufficient data to now report on skin biopsies conducted in Ontario for the first 38 weeks of 2020. We have included these data in our figures to show trends in biopsies beyond the first lockdown, and incorporated findings from these data into our results section, and the manuscript’s discussion. We hope that the reviewers and Editor will find this to their satisfaction, and provide sufficient information on our cohort.

We now state in our results that: ‘Following the precipitous drops in skin biopsies seen in the early lockdown (weeks 11-15), a slow increase in numbers of skin biopsies was observed over the following 10 weeks. However, following lockdown, skin biopsy rates in most weeks of 2020 did not meet those of the same week in 2019 (Figures 2a, 2b, 2c). As skin cancer incidence is expected to increase from year to year, this deficit is noticeable, with 45,710 fewer cases for all skin biopsies, 9104 for KC and 595 for melanoma between weeks 11-38 of 2020, compared to 2019 weeks 11-38.'

We have also incorporated Reviewer #2’s comments into our discussion and conclusion, for example:

‘In the absence of a diagnostic biopsy, it is difficult to ascertain the risk posed by a suspicious skin lesion; a lesion thought to be BCC could be melanoma. The impact of pandemic-related delay on skin cancer outcomes is yet unknown but is a cause for concern given the strong relationship between early detection and skin cancer outcomes. … Of great concern, we found that almost half a year after lockdown measures started, there were still large deficits in expected biopsy cases, with 45,710 less cases for all skin biopsies, 9104 for KC and 595 for melanoma between weeks 11-38 of 2020, compared to 2019 weeks 11-38. This does not take into account normal expected increases in cancer incidence year to year.’
